# WNT4/β-Catenin Pathway Maintains Female Germ Cell Survival by Inhibiting Activin βB in the Mouse Fetal Ovary

**DOI:** 10.1371/journal.pone.0010382

**Published:** 2010-04-29

**Authors:** Chia-Feng Liu, Keith Parker, Humphrey H.-C. Yao

**Affiliations:** 1 Department of Veterinary Biosciences, University of Illinois, Urbana-Champaign, Illinois, United States of America; 2 Department of Internal Medicine, University of Texas Southwestern Medical Center, Dallas, Texas, United States of America; Temasek Life Sciences Laboratory, Singapore

## Abstract

Female germ cells are essential for organogenesis of the ovary; without them, ovarian follicles do not form and functional and structural characteristics of the ovary are lost. We and others showed previously that when either *Wnt4* or β-catenin was inactivated in the fetal ovary, female germ cells underwent degeneration. In this study, we set out to understand whether these two factors belong to the same pathway and how they maintain female germ cell survival. We found that activation of β-catenin in somatic cells in the *Wnt4* knockout ovary restored germ cell numbers, placing β-catenin downstream of WNT4. In the absence of *Wnt4* or β-catenin, female germ cells entered meiosis properly; however, they underwent apoptosis afterwards. Activin βB (*Inhbb*), a subunit of activins, was upregulated in the *Wnt4* and β*-*catenin knockout ovaries, suggesting that *Inhbb* could be the cause for the loss of female germ cells, which are positive for activin receptors. Indeed, removal of *Inhbb* in the *Wnt4* knockout ovaries prevented female germ cells from undergoing degeneration. We conclude that WNT4 maintains female germ cell survival by inhibiting *Inhbb* expression via β-catenin in the somatic cells. Maintenance of female germ cells hinge upon a delicate balance between positive (WNT4 and β-catenin) and negative (activin βB) regulators derived from the somatic cells in the fetal ovary.

## Introduction

Female germ cells not only are essential for the propagation of species but also play an important role in ovarian organogenesis. Presence of germ cells is required for the formation of follicles, the functional unit of the ovary. Disruption of germline-specific genes, such as *Dazla* and Factor in the germline α (*Figla*) led to degeneration of oocytes and failure in folliculogenesis [1], [2], [3]. In addition, if germ cells are lost after follicle formation, characteristics of the ovary vanish [4], [5], [6]. Therefore, defects in female germ cell survival have detrimental impacts on fertility and reproductive health of the affected individuals.

Once they have migrated to the gonad, germ cells start to differentiate by following their intrinsic programs as well as responding to instructions from the somatic environment [7], [8]. In mouse fetal ovary, female germ cells enter meiosis around 14.5 dpc (day post coitum) as a result of the action of mesonephros-derived retinoic acids [9], [10] and then immediately arrest at the prophase of meiosis I [8], [11]. Ideas of the influences of somatic cells on female germ cell development have long been proposed. Gene screening schemes have yielded putative candidate genes that may play roles in this process. Among these candidates, the Wingless-type MMTV integration site (*Wnt*) family of genes, including *Wnt4, Wnt5a, Wnt6*, and *Wnt9a*, are found expressed in the somatic cells of the fetal ovary [12], [13]. WNT proteins are known to be involved in cell fate decision and cell cycle regulation [14]. These ovarian WNTs may work synergistically or redundantly in regulating female germ cell development. Previous studies by our lab and others have shown that inactivation of *Wnt4* or β-catenin resulted in degeneration of female germ cells starting at 16.5 dpc [15], [16], [17], [18]. In this study, we provide evidence that β-catenin lies downstream of WNT4 to suppress expression of activin βB. When the Wnt4/β-catenin pathway is inactivated, upregulation of activin βB leads to loss of female germ cells.

## Results

### Effects of somatic cell-specific inactivation of β-catenin on female germ cell apoptosis and meiosis

**Figure 1:**
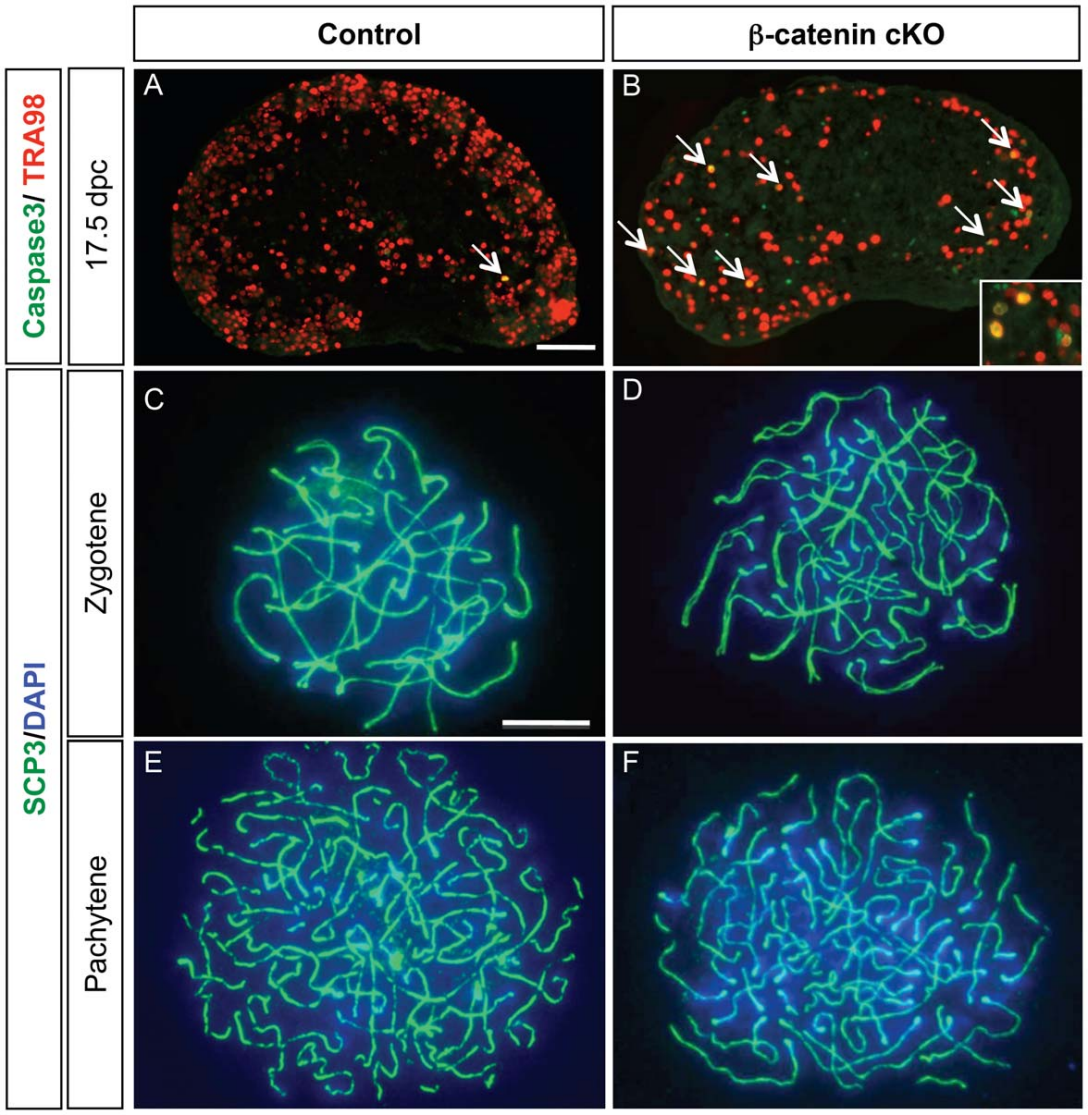
Effects of somatic cell ablation of β-catenin on germ cell apoptosis and entry into meiosis. (A–B) Immunohistochemistry for germ cell marker TRA98 (red) and apoptotic marker cleaved caspase 3 (green) in control (*SF1/cre;Ctnnb1^f/+^*) and β-catenin conditional KO (cKO; *SF1/cre;Ctnnb1^f/−^*) ovaries at 17.5 dpc. The arrows indicate cells that are double-positive (yellow) for TRA98 and cleaved caspase 3. The inset in B is an image of a higher magnification of cells double-positive (yellow) for TRA98 and cleaved caspase 3. (C–F) Analysis of the meiotic status of germ cells via immunohistochemistry for SCP3 on germ cell smear obtained from the control and β-catenin conditional KO ovaries at 15.5 dpc. The samples were counterstained with nuclear DAPI (blue). Scale bar represent 100 µm in A–B and 10 µm in C–F.

In our previous study, we generated a somatic cell-specific β-catenin conditional knockout (cKO) mouse by introducing the Steroidogenic factor 1-cre (SF1/cre) transgene into an embryo carrying floxed and null β-catenin alleles (*Ctnnb1^f^* ^/−^) [18]. The SF1/cre mouse line starts to show Cre recombinase activity in the somatic cells of fetal gonads around 10.5–11.5 dpc [19]. The Cre recombinase removes the DNA sequence between the two *loxP* sties that flank the β-catenin gene, therefore producing a null allele of β-catenin. Inactivation of β-catenin in the SF1-positive somatic cells of fetal ovaries resulted in a progressive loss of female germ cells starting at 16.5 dpc [18]. Double staining of the germ cell marker TRA98 and the apoptotic marker cleaved caspase 3 revealed an increase in germ cell apoptosis in the β-catenin cKO ovaries compared to the control (*SF1/cre; Ctnnb1^f/+^* or *Ctnnb1^f/−^*) starting at 17.5 dpc (Fig. 1A–B). On average, only one or two germ cells underwent apoptosis per section in control ovaries (Fig. 1A) but more than 5 apoptotic germ cells per section were observed in the β-catenin cKO ovaries (Fig. 1B), indicating that β-catenin in the SF1-positive somatic cells is involved in regulation of germ cell apoptosis.

Defects in the meiotic machinery are a major cause for loss of female germ cells [20]. To evaluate whether progression of meiosis was compromised in the absence of β-catenin, we performed immunostaining for synaptonemal complex protein 3 (SCP3) on chromosome smear obtained from female germ cells at 15.5 dpc and 16.5 dpc. Staining of SCP3, a scaffold protein formed on prophase I of meiosis, allowed us to monitor the progression of meiosis in female germ cells. In the absence of β-catenin, most female germ cells entered and progressed through prophase I of meiosis (zygotene and pachytene stages according to [21]) indistinguishable from the control (Fig. 1C–F). We previously analyzed the presence of another meiotic marker, phosphorylated-Histone2AX to detect double strand breaks in the DNA recombination events and found no differences between control and β-catenin cKO ovaries [18]. These findings together indicate that β-catenin in the somatic cells of fetal ovaries is not required for meiosis entry and progression of female germ cells; however, β-catenin is indispensable in the SF1-positive somatic cells for meiotic germ cell survival.

### Establishment of the connection between β-catenin and *Wnt4* in female germ cell survival

The germ cell loss phenotype in the β-catenin cKO ovary shares striking similarities with that in the *Wnt4* KO ovary [16], [18], [22], suggesting these two factors belong to a common pathway. To test whether β-catenin is a downstream mediator of WNT4, we introduced a constitutively active form of β-catenin (*Ctnnb1^fl.(ex3)^*) specifically in the SF1-positive somatic cells in the *Wnt4* KO ovary. *Ctnnb1^fl.(ex3)^* mice contain a genetically engineered β-catenin gene that *loxP* sequences are inserted in either side of the exon 3. The peptide encoded by the exon 3 is responsible for degradation of β-catenin. Once the exon 3 is removed by the Cre recombinase, the mutant β-catenin becomes resistant to degradation and therefore constitutively active in the SF1-positive somatic cells [23].

**Figure 2:**
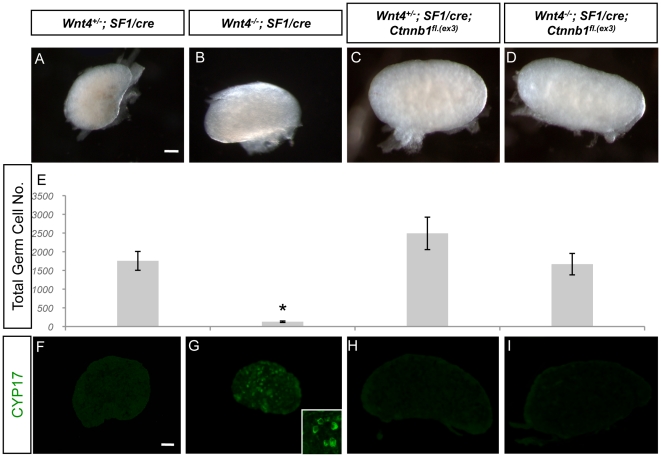
Effects of constitutively active form of β-catenin on female germ cell survival in the *Wnt4* KO ovary. (A–D) Whole mount light field images of *Wnt4* ^+/−^;*SF1/cre, Wnt4* ^−/−^;*SF1/cre*, *Wnt4^+/−^*;*SF1/cre; Ctnnb1^fl.(ex3)^*, and *Wnt4^−/−^;SF1/cre; Ctnnb1^fl.(ex3)^* ovaries at birth. (E) Average total germ cell number was obtained from *Wnt4^+/−^;SF1/cre*, *Wnt4^−/−^*;*SF1/cre*, *Wnt4^+/−^;SF1/cre*; *Ctnnb1^fl.(ex3)^*, and *Wnt4^−/−^;SF1/cre; Ctnnb1^fl.(ex3^* ^)^ ovaries at birth (n = 3 embryos for each genotype). Tukey tests revealed that the average germ cell number in *Wnt4^−/−^;SF1/cre* was significantly different from that in *Wnt4* ^+/−^;*SF1/cre* (P = 0.017), *Wnt4^+/−^;SF1/cre; Ctnnb1^fl.(ex3)^* (P = 0.002), and *Wnt4^−/−^;SF1/cre*; *Ctnnb1^fl.(ex3)^* (P = 0.023). The asterisk represents statistical significance. (F–I) Immunohistochemical staining for androgen-producing enzyme CYP17 (green) was performed in *Wnt4* ^+/−^;*SF1/cre*, *Wnt4* ^−/−^;*SF1/cre*, *Wnt4^+/−^*;*SF1/cre; Ctnnb1^fl.(ex3)^*, and *Wnt4^−/−^;SF1/cre; Ctnnb1^fl.(ex3)^* ovaries at birth. The inset in G represents a higher magnification of the CYP17-positive cells. Scale bar = 100 µm.

We hypothesized that if β-catenin is a downstream effecter of WNT4, introducing active β-catenin to the *Wnt4* knockout ovaries should restore normal germ cell development. We examined the total germ cell numbers in the newborn ovaries from controls (*Wnt4^+/−^*;*SF1/cre* and *Wnt4^+/−^;SF1/cre;Ctnnb1^fl.(ex3^* ^)^), *Wnt4* KO (*Wnt4^−/−^*;*SF1/cre*), and *Wnt4* KO plus active β-catenin (*Wnt4^−/−^;SF1/cre;Ctnnb1^fl.(ex3^* ^)^; Fig. 2A–D). To obtain the total germ cell number per ovary, we sectioned the entire ovary, stained the sections with germ cell marker TRA98, and counted TRA98-positive germ cells in sections that were 30 µm apart. The total germ cell number in the *Wnt4* KO ovary was significantly lower than the controls (Fig. 2E), consistent with previous findings [22], [24]. However, presence of active β-catenin in the *Wnt4* KO ovaries increased the total germ cell numbers to the level similar to the controls (Fig. 2E). Although the size of ovaries in the female with active β-catenin (Fig. 2C & D) was larger than that in the female without the active β-catenin (Fig. 2A & B), the difference in ovary size did not contribute to the difference in total germ cell numbers.

In addition to the restoration of female germ cells, the ectopic CYP17-positive cells in the *Wnt4* KO ovary (Fig. 2G) were no longer present in the *Wnt4^−/−^; SF1/cre; Ctnnb1^fl.(ex3)^* ovary (Fig. 2I), indicating activation of β-catenin in SF1-positive *Wnt4* KO somatic cells were able to prevent the ectopic appearance of CYP17-postve cells. This genetic evidence places β-catenin downstream of WNT4 in a somatic cell-specific pathway responsible for female germ cell survival and preventing ectopic appearance of CYP17-positive cells in the fetal ovary.

### Exclusion of the involvement of androgens in germ cell loss phenotype in the β-catenin cKO ovary

**Figure 3:**
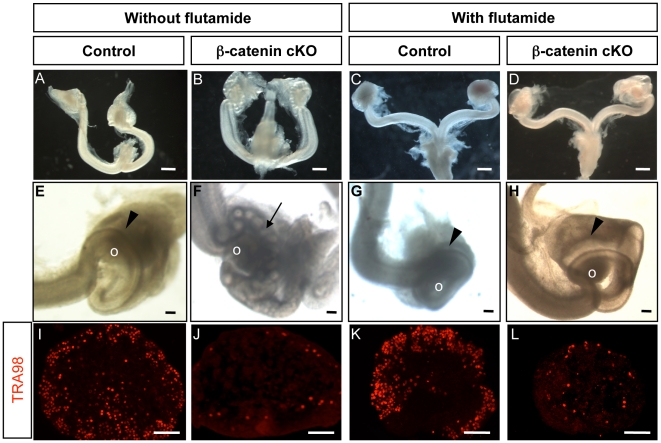
Effects of flutamide treatment on development of the reproductive tract and germ cells in β-catenin conditional KO female. (A–H) Whole mount images of reproductive tracts and ovaries and (I–L) immunohistochemistry for TRA98 were performed on the ovary of the control (*SF1/cre;Ctnnb1^f/+^*) and β-catenin cKO (*SF1/cre;Ctnnb1^f/−^*) female with or without flutamide treatment. o =  ovary; arrow =  epididymis, arrowhead =  oviduct. Scale bar represents 500 µm in A–D and 100 µm in E–L.

In addition to germ cell loss, inactivation of *Wnt4* or β-catenin resulted in ectopic appearance of androgen-producing CYP17-positive cells in the ovary (Fig. 2F & G) [18], [25]. These ectopic CYP17-positive cells produce sufficient androgen that maintains androgen-dependent male reproductive organs such as epididymis and vas deferens in the *Wnt4* and β-catenin cKO female embryos [18], [22]. To examine whether ectopic androgen production is responsible for the loss of germ cells, we injected the anti-androgen flutamide daily from 12.5 dpc to birth into pregnant female mice carrying β-catenin cKO embryos. Flutamide is a potent androgen antagonist that has been wildly used to block androgenic effects for clinical treatment of prostate cancer and for basic research on androgen action during embryogenesis [26]. Flutamide injection efficiently blocked the masculinizing effects of androgens in the β-catenin cKO female embryos based on the fact that male reproductive characteristics such as the epididymis were inhibited (Fig. 3D & H) compared to the cKO female without flutamide treatment (Fig. 3B & F). To further confirm that androgen functions were properly inhibited, we examined control male embryos exposed to flutamide in utero. We observed underdeveloped testis and other male reproductive organs compared to the vehicle-treated control (data not shown). These results were similar to what was reported in the literature [27], indicating that the flutamide treatment was sufficient to block androgen action in our system. However, regardless the presence or absence of flutamide treatment, loss of female germ cells was still observed in the β-catenin cKO ovaries at birth (Fig. 3J & L). Flutamide treatment had no effects on development of the female reproductive systems and female germ cells in the control females (*SF1/cre; Ctnnb1^f/+^*; Fig. 3A, C, E, G, I, & K). These results demonstrate that loss of germ cells in the β-catenin cKO ovary does not result from ectopic androgen production.

### Genetic identification of activin βB as the factor downstream of WNT4/β-catenin that is responsible for inducing female germ cell loss

**Figure 4:**
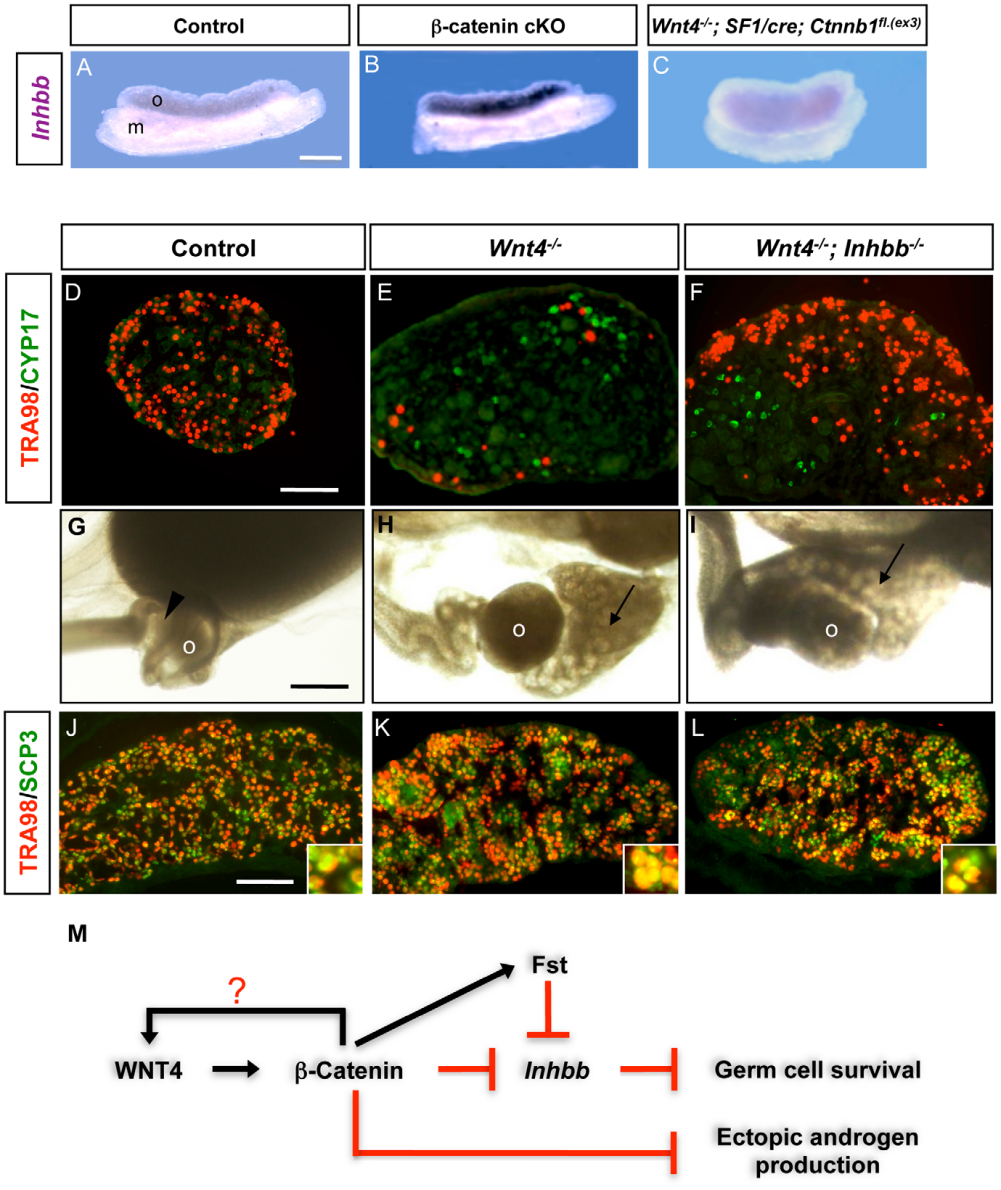
Involvement of *Inhbb* in germ cell loss in the absence of *Wnt4*. (A–C) Whole mount in situ hybridization for *Inhbb* was performed on control ovary (*SF1/cre;Ctnnb^f/+^* or *Ctnnb1^f/−^*) (A), β-catenin cKO ovary (*SF1/cre;Ctnnb^f/−^*) (B), and *Wnt4^−/−^;SF1/cre; Ctnnb1^fl.(ex3)^* ovary (C) at 13.5 dpc. (n = 2–3 for each genotypes). o =  ovary, m =  mesonephros. (D–F) Immunohistochemistry for TRA98 (red) and CYP17 (green) was performed on ovary sections from control (*Wnt4^+/−^; Inhbb^+/−^*), *Wnt4* single KO and *Wnt4; Inhbb* double KO ovary at birth. (G) Light field microscopic images of the reproductive tract were taken from control female, *Wnt4* single KO, and *Wnt4; Inhbb* double KO females at birth. Arrowheads indicate oviduct and arrows indicate epididymal structure. o = ovary. (J–L) Immunohistochemistry for TRA98 (red) and SCP3 (green) were performed on ovary sections from control, *Wnt4* single KO and *Wnt4; Inhbb* double KO ovary at 15.5 dpc. At this stage, the female germ cells have not lost yet in the *Wnt4* KO ovaries, allowing us to monitor the status of meiosis. The insets are the images of a higher magnification of cells double-positive (yellow) for TRA98 and SCP3. Scale bar represent 250 µm in A–C, G–I and 100 µm in D–F, J–L. (M) A proposed model for the somatic cell-derived pathway on female germ cell survival: In the mouse fetal ovary, WNT4 signals via β-catenin to decrease the expression of activin βB or *Inhbb*, which causes loss of meiotic germ cell. WNT4 also stimulates the production of follistatin (*Fst*), which acts to antagonize the activity of *Inhbb*. The WNT4/β-catenin pathway also prevents the ectopic production of androgens in the fetal ovary, which is not responsible for the germ cell loss. WNT4 could possibly regulate its own expression via β-catenin.

The TGFβ superfamily has been shown to play a role in inducing apoptosis [28], [29]. In the case of freemartins, where female embryos were exposed to anti-Müllerian hormone, a member of the TGFβ superfamily, germ cell loss was observed [30], [31]. Therefore, we screened various TGFβ family members that showed an increased expression in the fetal ovary lacking either *Wnt4* or β-catenin. We found that mRNA expression of activin βB (*Inhbb*) was significantly elevated in the *Wnt4* KO ovaries [32] and β-catenin cKO ovary compared to the control (Fig. 4A–B). In addition, introduction of the active β-catenin to the *Wnt4* KO ovary decreased *Inhbb* mRNA expression to the level similar to that in the control (Fig. 4C). We therefore hypothesized that if elevated *Inhbb* is indeed responsible for female germ cell loss in the absence of *Wnt4*, removal of *Inhbb* in the *Wnt4* KO background should reverse this phenotype. Indeed, in the *Wnt4* ^−/−^; *Inhbb* ^−/−^ double KO ovary, female germ cell number was significantly increased compared to the *Wnt4* single KO at birth (Fig. 4D–F, n = 3). To monitor the status of germ cell meiosis, we examined the expression of SCP3, a meiosis marker, in control, *Wnt4* single knockout, *and Wnt4* ^−/−^; *Inhbb* ^−/−^ double KO ovaries at 15.5 dpc the time before the germ cell demise occurred in the *Wnt4* KO ovary. Female germ cells in the *Wnt4* ^−/−^; *Inhbb* ^−/−^ double KO ovary entered meiosis properly as evident by double immunostaining for SCP3 and TRA98 (Fig. 4J–L). Similar to the *Wnt4* single knockout ovary, ectopic CYP17-positive cells and epididymal structure were found in the *Wnt4* ^−/−^; *Inhbb* ^−/−^ double KO ovary but were absent in the control female at birth (Fig. 4G–I), further supporting that ectopic production of androgen is not responsible for the loss of female germ cells.

## Discussion

The WNT4/β-catenin pathway, operating in the SF1-positive somatic cells in fetal ovaries, is essential for maintaining the survival of meiotic germ cells. Although the initiation and progression of meiosis are not affected by the absence of WNT4/β-catenin in the fetal ovary, the meiotic germ cells undergo apoptosis and are lost at birth. WNT4 maintains female germ cell survival by activating β-catenin in the SF1-positive cells, which in turn suppresses expression of activin βB. Our results provide the genetic proof of somatic cell contribution to female germ cell survival via a delicate balance between positive (Wnt4 and β-catenin) and negative regulators (activin βB or *Inhbb*, Fig. 4M).

Somatic cells of the fetal ovary are the supporting cells that nurture the germ cells and provide them proper environment to grow. *In vitro* experiments using human and mouse ovarian tissues demonstrated that factors such as Kit ligand, leukemia inhibiting factor, bone morphogenetic factor 4, basic fibroblast growth factor, and activin A stimulate folliculogenesis and survival of germ cells in culture [33], [34]. However, *in vivo* evidence is lacking to support a functional role of these factors in female germ cell development in the fetal ovary. In the *Wnt4* knockout and β–catenin cKO ovary, female germ cells undergo apoptosis and are progressively lost. Despite entering meiosis properly, most germ cells disappear around the time of birth. The ability of constitutively active β-catenin to restore germ cell numbers in the *Wnt4* KO ovary indicates that β-catenin is involved directly or indirectly in the downstream pathway of WNT4. Evidence of WNT4 signaling via β-catenin is also found in nephron induction, kidney epithelial cells, and renal fibrosis [35], [36], [37], [38]. These observations collectively support the model that β-catenin operates downstream of WNT4 in the fetal ovary. In addition to serving as an intracellular signaling molecule of WNT4, β-catenin also has a possible role in regulating the expression of *Wnt4*. We found that expression of *Wnt4* was lost in the absence of β-catenin in the SF-1 positive-somatic cells of the fetal ovary; however, R-spondin 1 (*Rspo1*) expression was not altered [18]. These results suggest that RSPO1 or other WNT proteins including WNT4 itself could stimulate *Wnt4* expression via β-catenin.

Ovaries with active β-catenin in the somatic cells are larger than the controls, suggesting that the activation of β-catenin promotes proliferation of somatic cells. WNT/β-catenin signaling pathway is known to regulate genes that are involved in cell proliferation and cell fate decision during embryogenesis [14]. Mis-regulation of WNT/β-catenin signaling pathway results in various types of cancers [39], [40], [41], [42]. Introduction of activated β-catenin to fetal testes also increases the size of affected testis [43]. Further experiments are needed to investigate the impact of active β-catenin on somatic cell proliferation.

Germ cell loss still occurred in *Wnt4* KO and β-catenin cKO fetal ovaries after anti-flutamide treatment, therefore excluding the involvement of androgens. Furthermore, androgen receptors are not present in germ cells at fetal stages [44], supporting our conclusion that ectopic androgen is not responsible for the death of germ cell in *Wnt4* KO and β-catenin cKO fetal ovaries. Rescue of the germ cell loss phenotype in the *Wnt4^−/−^*; *Inhbb^−/−^* double KO ovary provides a genetic link implicating *Inhbb* as the gene responsible for germ cell demise. *Inhbb* encodes the subunit for inhibin B and activin B. Germ cells are known to express receptors (Acrr-IB and ActR-IIB) for activins [45]. The ectopic production of activin B from somatic cells of the fetal ovary in the absence of *Wnt4* and β–catenin could therefore act directly on female germ cells and cause their death. Using Transcription Element Search System (TESS) program, we found several putative β-catenin LEF/TCF response elements in the promoter region of *Inhbb*. β-catenin could bind to these response elements and suppress the expression of *Inhbb* in the fetal ovary.

The germ cell loss phenotype is also observed in the follistatin (*Fst*) knockout fetal ovaries [24]. Based on a genetic epistasis experiment, *Fst* acts downstream of WNT4 [24]. Furthermore, expression of mouse *Fst* is dependent upon a consensus β-catenin LEF/TCF binding site in the promoter region [46], [47], and expression of *Fst* was lost in the β-catenin cKO ovary [18], placing *Fst* downstream of β-catenin. FST is known to bind activins with high affinity, therefore preventing activins from activating their receptors [48]. Expression of *Inhbb* mRNA is present in mouse gonads of both sexes at 11.5 dpc. Its expression is down-regulated but remains detectable in the ovary at 12.5 dpc [32]. Interestingly, in contrast to the *Wnt4* and β-catenin cKO ovary where *Inhbb* expression is upregulated, *Inhbb mRNA* expression levels are not altered in the absence of *Fst* [32]. We speculate that the function of FST is to antagonize and inhibit the action of the residual activin B to prevent it from affecting female germ cell survival. WNT4/β-catenin acts at two levels to block the effects of activin B on germ cells, by downregulating transcription of *Inhbb* and by activating FST to antagonize activin B protein (Fig. 4M).

It is known that oocytes start entering apoptosis prenatally, therefore leaving a finite number of oocytes for the rest of the reproductive life of female individuals. At least two hypotheses have been proposed for the cause of female germ cell demise during embryogenesis [49], [50]. The first possible mechanism is that abnormal oocytes with defects on their chromosomes or mitochondrial genomes are eliminated from the oocyte pool via intrinsic check-point mechanism [51]. Another possibility is that the somatic cell environment controls the numbers of female germ cells. In this study, we found that the upregulated of *Inhbb* resulted in the death of female germ cell in the absence of Wnt4/β-catenin signaling from somatic cells. We propose that the balance between somatic cell signaling (WNT4/β-catenin) and Activin B (the protein product of *Inhbb*) is critical for the maintenance of female germ cells during embryonic stage. It is possible that increasing germ cell apoptosis close to birth is the result of a shifted balance toward action of activin B. If our hypothesis is correct, one would predict that loss of *Inhbb* should lead to decrease in germ cell apoptosis and presumably more oocytes in the ovary. *Inhbb* knockout females are fertile despite an increase in length of gestation and a decrease in ability of nursing [52]. It remains to be determined whether more oocytes are present in the *Inhbb* knockout ovary.

When either *Wnt4* or β-catenin is inactivated, female germ cells enter meiosis and progress to meiosis prophase I normally, based on the time course analysis of chromosome smears of germ cells and examination of expression of SCP3 and *γ*H2AX [16], [17], [18], [24]. These findings suggest that the retinoic acid (RA) pathway that regulates meiosis entry is probably not affected by the absence of *Wnt4*/β-catenin. Studies on the R-spondin1 (*Rspo1*) KO mice show that *Rspo1* is the upstream regulator of WNT4 and β-catenin in ovarian development [15], [17]. In the absence of *Rspo1*, components of the RA pathway are not significantly affected [15]. Although a decrease in germ cell numbers is reported in the *Rspo1* KO ovary at 14.5 and 16.5 dpc, germ cell entry into meiosis appeared to be normal based on the SCP3 staining [17]. The possibility of a direct action of RSPO1 on female germ cells remains to be determined.

Involvement of WNT/β-catenin pathway in regulating proliferation of primordial germ cell (PGCs) is evident in *Drosophila* and mouse. Activation of β-catenin in PGCs promotes proliferation in *Drosophila* whereas it delays cell cycle progression in mouse [53], [54]. In this study, we report an essential while indirect role of β-catenin in somatic cells in controlling female germ cell numbers. β-catenin, operating downstream of WNT4 in the somatic cells, acts as a suppressor of activin βB, which is a negative regulator of female germ cell survival. In summary, our data provide genetic identification of a molecular hierarchy in ovarian somatic cells that is essential for maintenance of female germ cell survival.

## Materials and Methods

### Ethics Statement

All procedures described were reviewed and approved by the Institutional Animal Care and Use Committee at University of Illinois, and were performed in accordance with the Guiding principles for the Care and Use of Laboratory Animals.

### Animals

*SF1/cre;Ctnnb1^floxed/−^* embryos were derived from breeding of the *Ctnnb1^+/−^*; SF1/cre, which carrying Cre recombinase under the control of the *Sf1* promoter and its regulatory elements [19], and the *Ctnnb1^floxed/floxed^* parental strains. *Wnt4^+/−^* mice were obtained from Jackson Laboratory (strain 129-*Wnt4* ^tm1Amc^/J). *Ctnnb1^fl(ex3)^* mice were obtained from Harada et al. [23]. To generate mice expressing the stabilized form of β-catenin specifically in the somatic cells of *Wnt4* knockout ovary, *Wnt4^+/−^; SF-1/cre* were mated with *Wnt4^+/−^*; *Ctnnb1^fl(ex3)^* mice to obtain *Wnt4^−/−^;Sf-1cre; Ctnnb1^fl(ex3)^* embryos. To obtain the *Wnt4* and Activin β B double knockout mice (*Wnt4^−/−^*; *Inhbb^−/−^*), *Wnt4^+/−^* mice were mated to *Inhbb^+/−^* mice to generate *Wnt4^+/−^*; *Inhbb^+/−^* double heterozygotes. *Wnt4^+^* ^/−^; *Inhbb^+/−^* double heterozygotes were mated to generate *Wnt4^−/−^*; *Inhbb^−/−^* double knockout mice. The day when the vaginal plug was detected in the mated female was considered as 0.5 dpc. Genotypes were determined by PCR using gene specific primers. The primers were: SF-1/Cre genotyping: 5′-GTGTGAACGAACCTGGTCGA AATCAGTGCG-3′ and 5′-GCATTACCGGTCGATGCAACGAGTGATGAG-3′; *Ctnnb* null allele: 5′-AATCACAGGGACTTCCATACCAG-3′ and 5′-GCCCAGCCTTAGCCCAACT-3′; Ctnnb1 wild type and floxed alleles: 5′-AAGGTAGAGTG ATGAAAGTTGTT-3′ and 5′-CACCATTGTCCTCTGTCTATTC -3′; *Wnt4* null and wild type alleles: 5′- CTG AGGAAGAGCAGGGTCAC -3′, 5-ATGGTCACC CCCATTTTACA -3′ and 5-TGGATGTGGAATGTGTGCGAG-3′; *Ctnnb1^fl(ex3)^* alleles 5′-GGTAGTGGTCCCTGCCCTTGA CAC-3′ and 5′-CTAAGCTTGGCT GGACGTAAACTC-3′; *Inhbb* null allele: 5′-CTTGGGTGGAGAGGC TATTC-3′ and 5′ - AAAGCTGATGATCTCGGAGACG - 3′; *Inhbb* wild type allele: 5′-ATGGTC ACGGCC CTG CGC AA-3′ and 5′-GCGGAT CCC TCT GCA AAG CTG ATG ATT TC-3′; *Sry* genotyping: 5′-TGAAGCTTTTGGCTTTGAG-3′ and 5′-CCGCTGCCAAATTCTTTGG-3′. All experiments were performed on at least two to five animals for each genotype.

### Immunohistochemistry

Samples were fixed in 4% paraformaldehyde overnight at 4°C and then washed in PBS for 5 minutes (3 times) Samples were put through a sucrose gradient (10%, 15% and 20%) and incubated in 1∶1 20% sucrose and OCT freezing media (Tissue-Tek) overnight at 4°C. Samples were embedded in 1∶3 20% sucrose and OCT mix and cut to 10 µm thick frozen sections. Sections were washed with PBS and then blocked in the blocking solution (5% heat-inactivated donkey serum and 0.1% Triton X-100 in PBS) for 1 h at room temperature. Primary antibodies were added to the blocking solution and incubated with sections at 4°C overnight. Sections were then washed with the washing solution (1% heat inactivated donkey serum and 0.1% Triton X-100 in PBS) followed by incubation in the blocking solution with the corresponding secondary anybodies. Sections were then washed with the washing solution and mounted with DAPI antifade reagent. The sources and dilution of primary antibodies were the rat monoclonal antibody against germ cell nuclear fraction (TRA98, 1∶000, a gift from H. Tanaka), the rabbit polyclonal antibody against cleaved caspases-3 (1∶200; Cell Signaling), and the rabbit polyclonal antibody against CYP17 (1∶100, a gift from B. Hales). All the secondary antibodies were purchased from Jackson Immunochemical and a 1∶200 dilution was used.

### Chromosome smear and immunostaining

Fetal germ cell chromosome smear and immunostaining were performed according to the protocol described in [55]. Briefly, ovaries from 15.5 dpc embryos were incubated in a 24-well dish with the hypoextraction buffer (15 mM Tris,pH 8.2, 50 mM sucrose, 20 mM citrate, 5 mM EDTA, pH 8.2, 0.5 mM DTT, 0.09 mg/ml PMSF, collagenase, 0.5 mg/ml) for at least 30 min. Then each ovary was placed in a 10 µl drop of 0.1 M sucrose on the slide and another 10 µl drop of sucrose was added. The ovaries were dispersed by repetitive pipetting. Cell suspension was then placed onto the slide coated with the fixative (0.1% paraformaldehyde, pH 9.2, 0.1% Triton-X 100). The slides were placed in a humid chamber for 4 h and then were gently washed three times (5 min each) in 1∶250 photo-flo (Kodak) in water. Slides were air-dried and stored in −20°C.

For immunostaining of spread chromatin, slides were washed three times (10 min each) in 10% antibody dilution buffer (10% donkey serum, 3% BSA, and 0.05% Triton-X in phosphate-buffered saline or PBS). Then slides were incubated with anti-SCP3 antibody (1∶500, Abcam) in a humid chamber overnight at 4°C. Samples were washed three times (10 min each) in 10% antibody dilution buffer. Slides were then incubated with secondary antibody for 2 h at room temperature in the dark followed by three washes (5 min each) in PBS. Slides were air-dried and mounted with DAPI antifade reagent.

### Germ cell counting

Newborn ovaries were obtained from 3 animals for each genotype. Samples were fixed in 4% paraformaldehyde in PBS at 4°C and processed according to the immunohistochemistry procedure described above. Germ cell count was obtained by counting TRA98-positive germ cells in sections (30 um apart) from the entire ovary. Data were analyzed using one-way ANOVA followed by Tukey test for pair wise comparisons.

### Flutamide treatments

Flutamide (F9397, Sigma-Aldrich) was dissolved in 1∶1 (vol/vol) mixture of absolute ethanol and sesame oil. Ten pregnant *Ctnnb1* ^floxed/floxed^ female mice that were plugged by *SF-1/cre*; *β-Ctnnb1^+/−^* male were injected daily with flutamide subcutaneously (100 mg/kg/daily) from 12.5 dpc until birth [27]. Five pregnant mice from the same breeding scheme were treated with the vehicle (sesame oil) from 12.5 dpc until birth (control group).

### Whole mount *in situ* hybridization

Tissues were fixed overnight in 4% paraformaldehyde in PBS at 4°C and dehydrated through a methanol gradient (25%, 50%, 70%, and 100%) in PTW (0.1% Tween20 in DEPC-PBS). Samples were stored in 100% methanol at −20°C up to 6 months. *In situ* hybridization was processed according to the standard non-radioisotopic procedure using digoxigenin-labeled RNA probes for *Inhbb*. Gonads with mesonephroi attached were rehydrated through a methanol gradient then wash with PTW. The rehydrated gonads were treated with proteinase K (10 mg/ml) at 37°C for 12 minutes and then post-fixed in 4% paraformaldehyde/0.1% glutaraldehyde at room temperature for 20 minutes. Samples were pre-hybridized in the hybridization buffer (5x SSC pH 5.0, 50% formamide, 0.1% CHAPS, 0.1% Tween20, 1 mg/ml Yeast tRNA, 50 µg/ml Heparin, and 5 mM EDTA pH 8.0) at 65°C for 1 hour. Then digoxigenin-labeled *Inhbb* RNA probe was added into the solution and samples were rotated in an oven at 65°C overnight (12–16 hours). On the Next day, samples were washing with pre-warmed hybridization buffer followed by washing with MABTL (5% MAB, 0.1% Tween20 and 0.05% Levamisole). Samples were incubated in 20% sheep serum in MABTL blocking solution at room temperature for 2 hours followed by incubating in alkaline phosphatase-conjugated anti-digoxigenin at 4°C overnight on a shaker. On the third day, after washed in MABTL three times for 1 hour each, samples were incubated in alkaline phosphates substrate in NTMTL (0.1 M NaCl, 0.01 M Tris-HCl pH 9.5, 0.05 M MgCl_2_, 1% Tween 20, 0.05% Levamisole) for color development.
